# From accommodation to inclusion: understanding barriers and facilitators in clinical dental education for Chilean students with hearing disabilities: a qualitative study

**DOI:** 10.3352/jeehp.2026.23.20

**Published:** 2026-06-29

**Authors:** Josefa P. Retamal-Martínez

**Affiliations:** Dental School, Faculty of Medicine, Pontificia Universidad Católica de Chile, Santiago, Chile; The Catholic University of Korea, Korea

**Keywords:** Dental education, Qualitative research, Education of persons with hearing disabilities, Dental students, Chile

## Abstract

**Purpose:**

This study aimed to describe inclusive mechanisms supporting dental students with hearing disabilities, focusing on facilitators of and barriers to participation in clinical education.

**Methods:**

A qualitative case study was conducted at a Chilean university. Data were collected through semi-structured interviews with 2 students with hearing disabilities, 3 clinical instructors, and 1 Chilean Sign Language interpreter who works at the university and supports dental students with hearing disabilities, as well as through direct observation. Data were analyzed using inductive thematic analysis. Participant validation was used to enhance credibility.

**Results:**

Facilitators were identified across pedagogical, technological, communicative, and relational domains. Pedagogical adaptations included early access to materials, visual resources, and flexible assessments. Technological tools such as real-time transcription supported communication and access to information. Alternative communication strategies included lip reading, written support, visual cues, and Chilean Sign Language. The presence of an interpreter was a key enabling factor. Relational support from faculty, peers, and family members also contributed substantially. Faculty flexibility and willingness to adapt were particularly important. Barriers were identified at structural, institutional, communicative, and attitudinal levels. Clinical environments presented physical and acoustic challenges, including noise, limited space, and complex interactions. Institutional responses were often reactive and involved limited planning. Communication barriers affected interactions with patients and peers, as well as academic literacy. Faculty reported limited training in inclusive education. Attitudinal barriers included misconceptions about disability and limited experience with disability.

**Conclusion:**

Inclusive mechanisms are present but insufficiently systematized. Strengthening institutional planning and structural adaptations is essential to ensuring equitable clinical education.

## Graphical abstract

[Fig f1-jeehp-23-20]

## Introduction

### Background/rationale

Inclusive education in higher education seeks to ensure equitable access, participation, and success for all students [1]. Despite advances in policy and institutional support, significant challenges remain [2,3], particularly in professional programs that involve clinical training [4-7]. In dental education, clinical environments introduce additional complexity because communication, real-time interaction, and patient care are central to training. Students with hearing disabilities face specific challenges in these clinical contexts, including communication barriers, environmental constraints, and limited pedagogical adaptations [8]. Although inclusion in higher education has been examined, limited evidence specifically addresses clinical dental training for students with hearing disabilities. Understanding how facilitators and barriers operate in these environments is essential for improving inclusive practices and supporting meaningful participation in clinical education [2,4].

### Objectives

This study aimed to describe facilitators and barriers affecting the inclusion of students with hearing disabilities in clinical dental education at a Chilean university.

## Methods

### Ethics statement

This study was approved by the Ethics Committee for Social Sciences, Arts, and Humanities of the Pontificia Universidad Católica de Chile (ID: 24111002). All participants provided informed consent. To reduce the risk of identification, direct quotations were not included.

### Personal characteristics of the research team

#### Interviewer/facilitator

The author conducted all interviews. When necessary, interviews were conducted with the assistance of a sign language interpreter. To ensure accuracy and minimize potential loss of meaning during interpretation, interview transcripts were reviewed and validated by participants before inclusion in the study.

#### Credentials

The researcher is a dentist (DDS), is a specialist in endodontics, and holds a master’s degree in education with a specialization in higher education.

#### Occupation

The researcher is a university faculty member at the School of Dentistry and was completing a master’s thesis in education, with a specialization in higher education, at the time of the study.

#### Gender

The researcher is female.

#### Experience and training

The researcher had 13 years of professional experience as a dentist and 11 years of experience as a university faculty member. At the time of the study, she held the academic rank of assistant clinical professor.

### Relationship with participants

#### Relationship established

A previous professional relationship existed with some academic participants, as the researcher was a faculty member within the School of Dentistry where the study was conducted. However, the researcher did not hold a position of authority or direct evaluative role in relation to these participants. For the remaining participants, no prior relationship existed before the study. Additionally, the researcher was not teaching the student participants either prior to or during the study period.

#### Participant knowledge of the interviewer

Participants were informed during recruitment and through the informed consent process that the researcher was a university faculty member and a master’s degree candidate conducting the study as part of her thesis in higher education. They were also informed that the purpose of the research was to explore facilitators and barriers affecting the inclusion of students with hearing disabilities in clinical dental education, with the aim of contributing to more inclusive educational practices in higher education.

#### Interviewer characteristics

The researcher had professional and academic interests in inclusive education, clinical dental training, and accessibility in higher education. Given her background as both a clinician and a university educator, the researcher recognized that her experiences and perspectives could influence data interpretation. The study was motivated by an interest in understanding how students with hearing disabilities experience clinical education and how institutional and pedagogical practices may facilitate or hinder inclusion.

### Theoretical framework and methodology

#### Methodological orientation

This study used a qualitative case study design with an inductive thematic analysis approach to explore facilitators and barriers affecting the inclusion of students with hearing disabilities in clinical dental education.

#### Theoretical framework

The study was informed by inclusive education and drew on both the social and medical models of disability to examine how individual experiences interact with institutional, pedagogical, and environmental factors in clinical education contexts.

### Participant selection

#### Sampling

Participants were selected using convenience sampling based on their direct involvement in or experience with the inclusion of students with hearing disabilities in clinical dental education.

#### Recruitment approach

Participants were recruited face-to-face.

#### Sample size

Six participants were included: 2 students with hearing disabilities, 3 clinical instructors, and 1 Chilean Sign Language interpreter.

#### Non-participation

No participants withdrew from the study after agreeing to participate. However, 1 initially planned participant group, consisting of significant others identified through snowball sampling, was ultimately not recruited because of participants’ time constraints and recruitment difficulties.

### Setting and context

#### Data collection setting

Data were collected in university settings associated with the School of Dentistry. Interviews were conducted in person.

#### Presence of non-participants

In interviews involving participants with hearing disabilities, a Chilean Sign Language interpreter was present when required to facilitate communication. No other non-participants were present during data collection.

#### Sample characteristics

The sample included 6 participants directly involved in clinical dental education at a Chilean university: 2 students with hearing disabilities, 3 clinical instructors, and 1 Chilean Sign Language interpreter.

Inclusion criteria varied by participant group. Students were required to be enrolled in the dental program and to have a hearing disability, as well as experience in preclinical or clinical training. Clinical instructors were required to have direct teaching experience with students with hearing disabilities in clinical or preclinical settings. The interpreter was required to have experience supporting students with hearing disabilities in the university context.

Participants were selected based on their direct involvement in inclusion processes within clinical dental education. Data collection was conducted during the first semester of 2025.

### Data collection

#### Interview guide

Semi-structured interview and observation guides were developed by the researcher based on the study objectives and informed by methodological recommendations from the qualitative research literature. To strengthen methodological rigor, the instruments underwent temporal triangulation and were reviewed and validated by 3 expert judges with experience in the field. Recruitment and data collection procedures followed all guidelines established by the institutional ethics committee.

#### Repeat interviews

No repeat interviews were conducted.

#### Audio/visual recording

Audio recordings were used during interviews with participant consent. No video recordings were performed.

#### Field notes

Field notes were taken during and after interviews and observational activities to document contextual information and emerging reflections relevant to the analysis.

#### Duration

Interviews lasted approximately 60 to 90 minutes, depending on the participant and the use of interpretation support.

#### Data saturation

Data saturation was not formally established because of the small and highly specific nature of the sample. However, sufficient depth and recurrence of themes were identified to address the study objectives.

#### Transcript validation

Interview transcripts were returned to participants for review, feedback, and correction before analysis.

### Data analysis

#### Number of data coders

Data coding and analysis were conducted by a single researcher, who performed open and axial coding and developed the thematic categories.

#### Coding framework

The coding process involved open and axial coding to identify patterns and develop thematic categories related to facilitators and barriers in clinical dental education. A structured coding framework was developed iteratively throughout the analysis. Because the entire study, including data collection and analysis, was conducted in Spanish, the coding framework and thematic categories presented in this manuscript were translated into English for publication (Dataset 1).

#### Theme identification

Themes were derived inductively from the data through an iterative process of open and axial coding. Categories and themes emerged progressively during analysis rather than being predefined before data collection.

#### Software

ATLAS.ti ver. 25.0 (ATLAS.ti Scientific Software Development GmbH) was used to organize, manage, and analyze qualitative data throughout the coding and thematic analysis process.

#### Participant validation

Preliminary interpretations and emerging findings were discussed face-to-face during the validation process to enhance credibility and confirm consistency with participants’ experiences.

## Results

### Quotations presented

Because the sample was small and highly identifiable, direct participant quotations were intentionally not included to protect confidentiality and reduce the risk of participant identification. This measure was considered particularly important within the ethical framework approved by the institutional ethics committee, given the specificity and limited size of the participant group.

### Data and findings consistent

The findings presented were consistent with the data collected through interviews and direct observation and were supported through triangulation and participant validation.

### Clarity of major themes

Major themes related to facilitators and barriers affecting the inclusion of students with hearing disabilities in clinical dental education were clearly identified and systematically presented.

### Clarity of minor themes

The analysis also considered diverse experiences and less recurrent themes, which contributed to a broader understanding of inclusion processes in clinical dental education contexts.

## Discussion

### Key results

#### Facilitators for inclusion in clinical dental education

Facilitators affecting the inclusion of students with hearing disabilities emerged across pedagogical, technological, communicative, relational, and sociocultural dimensions. Participants described multiple pedagogical adaptations that supported participation in preclinical and clinical learning environments, including early access to lectures and study materials, flexible assessments, extended clinical time, and visual teaching resources such as images, videos, and color-coded notes. Reduced student-to-instructor ratios and coordination between faculty members and interpreters also improved accessibility. Alternative communication strategies played a central role in supporting participation and comprehension. These strategies included lip reading, written communication, visual supports, Chilean Sign Language, and the use of transparent masks when possible. Faculty members described adapting their communication styles by maintaining visual contact, repeating instructions, and incorporating visual explanations to improve understanding. The collaborative development of dental terminology in Chilean Sign Language by students and interpreters also emerged as an important strategy for supporting clinical learning.

Technological resources represented another important facilitator. Participants described the use of real-time transcription tools, microphones with automatic captioning, tablets, digital applications, and social media platforms to support communication, learning, and autonomy in academic and clinical settings. Flexible assessment practices also facilitated inclusion, including additional time during examinations, modifications to evaluation formats, clarification of instructions in Chilean Sign Language with interpreter support, and grading approaches that prioritized conceptual understanding over grammatical accuracy in written Spanish.

Relational support emerged as a critical component of inclusion. Faculty characteristics such as empathy, patience, flexibility, and willingness to adapt teaching practices were highly valued by students. Participants described inclusive educators as professionals who provided emotional support, repeated explanations when needed, and actively sought accessible ways to communicate clinical content. Support networks involving peers, family members, partners, tutors, and institutional staff also contributed substantially to students’ academic continuity and well-being. Emotional support, academic assistance, and collaborative learning practices were frequently described as essential for navigating clinical education. The Chilean Sign Language interpreter occupied a particularly important role in both educational and clinical settings. Beyond interpretation, interpreters acted as communicative and pedagogical mediators, facilitating understanding among students, faculty members, peers, and patients. Their role also extended to tutoring and study support.

Participants also emphasized the importance of recognizing students with hearing disabilities as capable and autonomous individuals in clinical education. Faculty members and interpreters highlighted students’ responsibility, perseverance, adaptability, and commitment to professional training. The findings also reflected recognition of the broader social value of inclusive dental care, particularly the importance of training professionals who can communicate directly with Deaf patients in Chilean Sign Language and contribute to more accessible healthcare environments.

#### Barriers to inclusion in clinical dental education

Participants identified multiple barriers affecting inclusion in clinical dental education across structural, institutional, communicative, and attitudinal dimensions. Structural challenges included noise, limited physical space, inadequate lighting, and the complexity of interactions in clinical environments, all of which created difficulties for communication and learning. Participants also described the need for larger clinical cubicles, visual alert systems, and environmental adaptations to improve accessibility. Institutional barriers were frequently described as reactive rather than proactive. Participants reported insufficient planning time for inclusive strategies, lack of institutional coordination, and limited integration between faculty and inclusion support services. Inclusive practices often depended on individual initiatives rather than being systematically embedded in clinical education structures.

Communication difficulties represented one of the most significant barriers identified in the study. Participants described challenges in interactions with patients, peers, and faculty members, particularly in fast-paced and communication-intensive clinical environments. Difficulties related to academic literacy were also reported, including comprehension of lengthy texts, written examinations, and academic writing in Spanish, which was described as a second language for Deaf participants. Social interactions during clinical training also presented challenges. Participants described tensions related to coordination with clinical partners, requests for peer support, and concerns regarding fairness in the distribution of clinical work. Experiences of social isolation and discomfort when seeking assistance outside close social circles were also reported.

Faculty members frequently described feeling insufficiently prepared to teach students with hearing disabilities in clinical settings. Although participants expressed interest in learning inclusive strategies and Chilean Sign Language, they also reported uncertainty and anxiety regarding inclusive teaching practices. Participants additionally described experiences of prejudice, misconceptions, and discriminatory attitudes toward individuals with hearing disabilities. These included assumptions regarding reduced efficiency, concerns about clinical errors, exclusion from group feedback processes, and communication practices that relied heavily on inaccessible auditory cues. Some participants also reported concerns regarding discrimination from patients and peers in clinical environments.

### Interpretation

The findings suggest that inclusion in clinical dental education for students with hearing disabilities is shaped by the interaction between facilitating mechanisms and persistent systemic barriers. Pedagogical adaptations, assistive technologies, alternative communication strategies, and relational support promoted participation and accessibility in communication-intensive clinical environments.

However, structural, institutional, communicative, and attitudinal barriers continued to limit inclusion. Noise, spatial constraints, and complex real-time interactions created significant challenges, consistent with previous research on clinical education settings [8]. Institutional responses were often reactive, reflecting limited anticipatory planning and insufficient faculty preparation in inclusive education and sign language.

These findings support literature suggesting that barriers are embedded within educational systems rather than inherent to students themselves [2]. They also align with studies showing that Deaf and hard-of-hearing students frequently rely on individual coping strategies to compensate for insufficient accessibility measures [4].

### Comparison with previous studies

The findings of this study are consistent with previous research showing that inclusion in higher education is shaped by the interaction between structural, pedagogical, and relational factors. Similar to prior studies, the barriers identified in this research were associated with communication difficulties, insufficient institutional planning, inaccessible learning environments, and limited faculty preparation in inclusive education [2,8]. The importance of technological resources, communication adaptations, and support networks as facilitators was also consistent with previous literature.

In agreement with studies focusing on Deaf and hard-of-hearing students, participants in this study described the need to develop individual coping and adaptation strategies in response to insufficient accessibility measures [4]. Likewise, the communication-intensive nature of clinical education appeared to intensify barriers related to noise, overlapping interactions, and real-time communication demands, as previously described in clinical training settings [8].

A notable contribution of the present study is its specific focus on clinical dental education, an area that remains relatively underexplored in the literature. Unlike broader studies on disability in higher education, these findings highlight how communication barriers, clinical workflows, and patient interactions uniquely shape inclusion in dental training environments.

### Limitations

Small sample size and single-site design limit the generalizability of this study. The lack of quotations reduces narrative depth.

### Generalizability

The findings of this study have several implications for higher education institutions, particularly those involved in clinical training. First, they highlight the need to strengthen faculty development in inclusive pedagogical practices, especially in communication-intensive environments. Second, they underscore the importance of ensuring that accessibility measures are not only available but also consistently implemented across different courses and clinical settings.

Additionally, the results suggest that institutions should adopt a more proactive approach to inclusion, integrating accessibility into the design of clinical teaching rather than relying on reactive accommodations. This includes anticipating communication barriers, adapting teaching strategies, and fostering collaborative practices between students, faculty, and support services.

### Suggestions

Future research should explore inclusion in clinical education across multiple institutions and healthcare disciplines. Higher education institutions should strengthen proactive accessibility planning and faculty training in inclusive practices within clinical teaching environments.

### Conclusion

This study demonstrates that inclusion in clinical education for students with hearing disabilities is a multidimensional and dynamic process shaped by the interaction between structural conditions, pedagogical practices, and individual agency. Although students develop adaptive strategies to navigate barriers, these strategies often compensate for systemic limitations rather than reflect fully inclusive environments.

The findings highlight the need to move beyond accommodation-based models toward more integrated and anticipatory approaches that embed accessibility within the core design of clinical education. By doing so, higher education institutions can better support not only access but also meaningful participation and belonging for students with disabilities.

## Figures and Tables

**Figure f1-jeehp-23-20:**
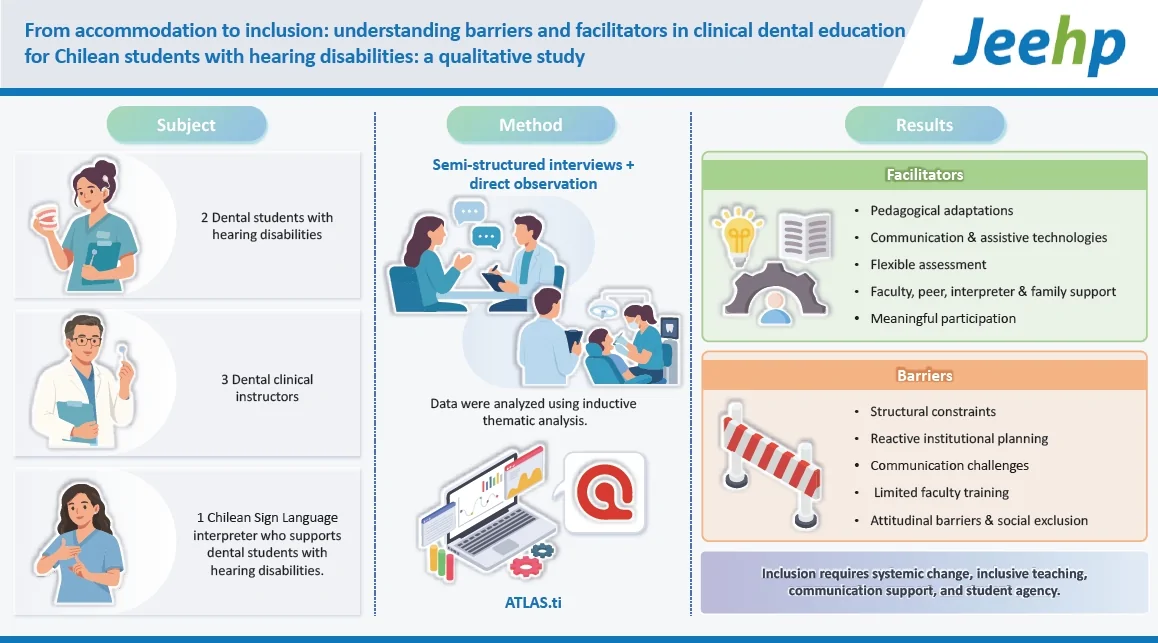


## Footnotes

**Data availability**
Data files are available from https://doi.org/10.7910/DVN/EFKJ5T Dataset 1. Coding framework.

## Supplementary materials

Supplement 1. Audio recording of the abstract.
