# Supplementary material for: From accommodation to inclusion: understanding barriers and facilitators in clinical dental education for Chilean students with hearing disabilities: a qualitative study
Source: J Educ Eval Health Prof. 2026 Jun 29;23:20. doi: 10.3352/jeehp.2026.23.20 (PMC13500177; doi:10.3352/jeehp.2026.23.20)
Supplement: Supplementary file 1 [file jeehp-23-20-dataset1.docx]

| **Category** | **Subcategory** | **Code** | **Description** |
| --- | --- | --- | --- |
| **Facilitators for the Inclusion of Students with Hearing Disabilities in Clinical Environments:** Refers to all factors, practices, and conditions identified by clinical faculty, the Chilean Sign Language interpreter, and students with hearing disabilities that promote equitable and meaningful participation in dental education contexts. | **Pedagogical, communicative, and technological adaptations:** This subcategory includes specific actions implemented in preclinical and clinical settings to adapt teaching-learning processes, communication, and assessment to the needs of students with hearing disabilities. It encompasses the use of images, subtitles, sign language, additional time, and digital support technologies, promoting educational equity and effective access to clinical content and experiences. Many of these supports are institutionally driven. | **Methodological adaptations and learning strategies** | Actions described include early access to lectures and study guides, reduced student-to-instructor ratios in practical settings, coordination with interpreters, respect for academic schedules, extended clinical time, and personal learning strategies such as recording lectures, transcription, simplified adaptations of complex texts, use of images, videos, and color-coded notes, as well as nighttime study routines. |
|  |  | **Alternative communication strategies** | Strategies mentioned include lip reading, the use of transparent masks when possible, speaking close to the cochlear implant, maintaining visual contact, repeating instructions, partial oral communication by the student, use of sign language, visual supports, drawings, illustrated guides, and subtitled videos. Willingness among clinical and educational teams to learn Chilean Sign Language was also highlighted. Adapted signs specific to dental terminology were collaboratively developed between the student and interpreter. |
|  |  | **Flexibility in assessment** | Adaptations included additional time during examinations, grading focused on content rather than language form, reduced clinical requirements, early initiation of assignments, changing assessment formats from audio to video, and clarification in Chilean Sign Language with interpreter support during tests and evaluations to facilitate comprehension and participation. |
|  |  | **Technological resources for inclusion** | Use of technologies such as microphones with automatic transcription, tablets with subtitles, and digital applications to facilitate communication and learning for students with hearing disabilities. Emerging tools and personal strategies, including social media and artificial intelligence, were explored to increase autonomy in academic and clinical contexts. |
|  | **Support figures within the academic environment:** This subcategory addresses the relational dimension of inclusive clinical education. It highlights the attitudes and practices of faculty, the central role of the interpreter as a communicative and pedagogical mediator, and the support provided by significant networks such as family members, peers, and tutors. These figures provide emotional, academic, and logistical support that enables more active and secure participation in clinical settings. | **Profile of the inclusive educator** | Characteristics described included empathy, respect, patience, clarity in explanations, use of visual supports, willingness to repeat content, emotional support, and close follow-up. Faculty reported limited previous experience teaching students with hearing disabilities, while students positively valued instructors who were demanding yet attentive and approachable. |
|  |  | **Significant individuals in academic support** | Various support figures were identified throughout students’ academic trajectories, including parents, partners, peers, and peer tutors. Specific supportive actions included emotional encouragement, study assistance, meal preparation, clarification of doubts, and support through WhatsApp groups. Institutional figures such as the student affairs coordinator or assigned student mentor were also highlighted. |
|  |  | **Role of the sign language interpreter** | The Chilean Sign Language interpreter played a central role in both clinical and educational settings. Their role extended beyond interpretation, facilitating bidirectional understanding, participating in tutoring sessions, supporting studying processes, and adapting to the challenges of clinical activities. |
|  | **Recognition of the student and the value of inclusive clinical education:** This subcategory reflects a transformative perspective on inclusion, where the student is not only granted accommodations but also recognized as an active individual capable of independently facing clinical challenges. It also emphasizes the ethical and symbolic importance of training professionals capable of providing accessible care to the Deaf community, positioning inclusion as both an educational and socially committed practice. | **Symbolic and social value of inclusive dental care** | Narratives emphasized the importance of accessible dental care for Deaf individuals, noting that direct care provided in Chilean Sign Language facilitates independence and autonomy among Deaf patients. Participation in inclusive clinical volunteer initiatives was also described. |
|  |  | **Recognition of student potential** | Faculty and interpreters valued and trusted the abilities of students with hearing disabilities. They recognized characteristics such as responsibility, perseverance, and proactive attitudes. Students expressed motivation, adaptation to clinical challenges, and determination to progress independently. Confidence and commitment to professional training were evident. |
|  |  |  |  |
| **Barriers to the Inclusion of Students with Hearing Disabilities in Clinical Environments:** Refers to limitations identified by clinical faculty, the Chilean Sign Language interpreter, and students with hearing disabilities that obstruct or hinder full access and participation in dental education experiences. | **Infrastructural and management-related interferences:** Includes difficulties associated with insufficient coordination, limited time, structural rigidity, and the absence of institutional clinical inclusion policies. | **Temporal and spatial adaptations in clinical work** | Needs identified included extended clinical time, larger clinical cubicles to accommodate interpreters, noise reduction, adequate lighting, use of mirrors, visual light alerts, and screens within clinical spaces. Difficulties related to clinical scheduling and administrative bureaucracy within the clinic were also described. |
|  |  | **Reactive institutional management and need for situated planning** | Participants described insufficient time to plan inclusive strategies within courses, the absence of a local coordinating body, and the need for contextualized planning within clinical environments developed collaboratively between faculty, students, and institutional inclusion programs. |
|  | **Communicative and clinical interaction obstacles:** Includes all difficulties that hinder effective communication and comprehension of oral and written content with patients, peers, and faculty members. | **Clinical care and communication with patients** | Challenges in communicating with hearing patients were described. Participants noted that the interpreter could sometimes limit rapport, as well as concerns related to medicolegal implications and fears associated with interacting with different patient profiles in the context of potential clinical errors. Faculty perceptions and student experiences in clinical settings were also discussed. |
|  |  | **Development of academic literacy** | Difficulties were described in written examinations, particularly open-ended assessments, comprehension of lengthy texts, and academic writing. Spanish was identified as a second language (sign leguage is first), and despite accommodations, persistent challenges continued to slow learning processes. |
|  |  | **Social interactions and distribution of clinical work with peers** | Difficulties were described regarding work organization and coordination with clinical partners, as well as tensions related to fairness in available time. Initial social isolation from peers was reported, although this improved with interpreter support. Participants expressed a preference for choosing their clinical partner and reported embarrassment when requesting help from individuals outside their close social circles. |
|  | **Attitudinal and sociocultural limitations:** Refers to beliefs, prejudices, and lack of knowledge regarding hearing disabilities that generate symbolic, emotional, or social exclusion within clinical and educational settings. | **Faculty training, awareness, and tensions regarding inclusion** | Faculty described feelings of insufficient preparation, interest in learning Chilean Sign Language and inclusive strategies, tensions between equity and equality, anxiety related to inclusion, and experiences without previous reference points. General training initiatives were mentioned, although participants emphasized the need for education in Deaf culture and identified limitations in methodological adaptation within clinical settings. |
|  |  | **Prejudices, perceptions of discrimination, and differential treatment toward individuals with hearing disabilities** | Participants described prior perceptions regarding the presumed difficulty or slowness of working with students with hearing disabilities. Initial fears toward the unfamiliar and beliefs that faculty, students, and patients might discriminate against students with disabilities due to assumptions that they require more time were also reported. Experiences of rejection by patients were described. Additional exclusionary practices included omitting explanations, physically relocating the student without notice, excluding the student from group feedback sessions, or relying on inaccessible auditory cues. |
